# Genome-wide association studies of toxicity to oxaliplatin and fluoropyrimidine chemotherapy with or without cetuximab in 1800 patients with advanced colorectal cancer

**DOI:** 10.1002/ijc.33739

**Published:** 2021-07-31

**Authors:** Katie Watts, Christopher Wills, Ayman Madi, Claire Palles, Timothy S. Maughan, Richard Kaplan, Nada A. Al-Tassan, Rachel Kerr, David Kerr, Victoria Gray, Hannah West, Richard S. Houlston, Valentina Escott-Price, Jeremy P. Cheadle

**Affiliations:** 1 Division of Cancer and Genetics, School of Medicine, Cardiff University, Cardiff, UK; 2 The Clatterbridge Cancer Centre NHS Foundation Trust, Bebington, UK; 3 Institute of Cancer and Genomic Sciences, Institute of Biomedical Research, University of Birmingham, Birmingham, UK; 4 CRUK/MRC Oxford Institute for Radiation Oncology, University of Oxford, Oxford, UK; 5 MRC Clinical Trials Unit, University College of London, London, UK; 6 Department of Genetics, King Faisal Specialist Hospital and Research Center, Riyadh, Saudi Arabia; 7 Department of Oncology, University of Oxford, Oxford, UK; 8 Nuffield Department of Clinical Laboratory Sciences, University of Oxford, John Radcliffe Hospital, Oxford, UK; 9 Division of Genetics and Epidemiology, The Institute of Cancer Research, London, UK; 10 Institute of Psychological Medicine and Clinical Neurosciences, School of Medicine, Cardiff University, Cardiff, UK

**Keywords:** chemotherapy, colorectal cancer, GWAS, toxicity

## Abstract

Chemotherapies administered at normal therapeutic dosages can cause significant side-effects and may result in early treatment discontinuation. Inter-individual variation in toxicity highlights the need for biomarkers to personalise treatment. We sought to identify such biomarkers by conducting 40 genome-wide association studies, together with gene and gene set analyses, for any toxicity and 10 individual toxicities in 1800 patients with advanced colorectal cancer treated with oxaliplatin and fluoropyrimidine chemotherapy ± cetuximab from the MRC COIN and COIN-B trials (385 patients received FOLFOX, 360 FOLFOX + cetuximab, 707 XELOX and 348 XELOX + cetuximab). Single nucleotide polymorphisms (SNPs), genes and gene sets that reached genome-wide or suggestive significance were validated in independent patient groups. We found that *MROH5* was significantly associated with neutropenia in MAGMA gene analyses in patients treated with XELOX (*P* = 6.6 × 10^−7^) and was independently validated in those receiving XELOX + cetuximab; pooled *P* = 3.7 × 10^−7^. rs13260246 at 8q21.13 was significantly associated with vomiting in patients treated with XELOX (odds ratio = 5.0, 95% confidence interval = 3.0-8.3, *P* = 9.8 × 10^−10^) but was not independently replicated. SNPs at 139 loci had suggestive associations for toxicities and lead SNPs at five of these were independently validated (rs6030266 with diarrhoea, rs1546161 with hand-foot syndrome, rs9601722 with neutropenia, rs13413764 with lethargy and rs4600090 with nausea; all with pooled *P*’s < 5.0 × 10^−6^). In conclusion, the association of *MROH5* with neutropenia and five other putative biomarkers warrant further investigation for their potential clinical utility. Despite our comprehensive genome-wide analyses of large, well-characterised, clinical trials, we found a lack of common variants with modest effect sizes associated with toxicities.

## 1 Introduction

Many patients diagnosed with colorectal cancer (CRC) receive chemotherapy either as part of their treatment for curative disease or to extend survival.^1^ Most chemotherapeutic agents are associated with significant side effects even if administered at normal therapeutic dosages.

The combination of fluoropyrimidine and oxaliplatin is a common first-line treatment for many cancers including CRC.^2^ XELOX (XEL = capecitabine, OX = oxaliplatin) is an oral fluoropyrimidine with similar efficacy to FOLFOX (FOL = folinic acid, F = fluorouracil, OX = oxaliplatin) but with differing toxicity profiles.^3,4^ Whereas XELOX often causes gastrointestinal symptoms and hand-foot syndrome, FOLFOX tends to affect immunity. Cetuximab, a monoclonal antibody directed against the epidermal growth factor receptor, is also used in the treatment of CRC and often causes skin rashes.^5^

Some toxicities have short-term acute effects whereas others remain after treatment has stopped.^6^ Toxicity adversely affects a patient’s quality of life and can be life threatening. Drug toxicity may result in treatment discontinuation or dose reduction,^7,8^ thus significantly affecting the prospects of a cure.^9,10^

Since there is significant inter-individual variation in chemotherapy-related toxicity, the identification of predictive biomarkers is highly desirable to personalise therapy. The role of inherited genetic factors is increasingly being recognised to influence patient chemotherapy-related toxicity. Notably, rare variants in the gene encoding dihydropyrimidine dehydrogenase (*DPYD*) are well established to be associated with severe toxicities to 5-fluorouracil (5-FU).^11,12^ While the role of common genetic variation is less clear, we and others have shown that common variants in *DPYD* also appear to affect the toxicity.^13–15^ To date, most studies have sought to identify inherited predictive biomarkers using candidate gene and variant-based analyses, based on preconceptions as to probable biology and using small cohorts of patients with no independent validation. To address such limitations, we have analysed genome-wide association study (GWAS) data on 1800 patients with advanced CRC treated with oxaliplatin and fluoropyrimidine chemotherapy ± cetuximab with replication in independent patient groups.

## 2 Materials and Methods

### 2.1 Patients and samples

**Figure 1:**
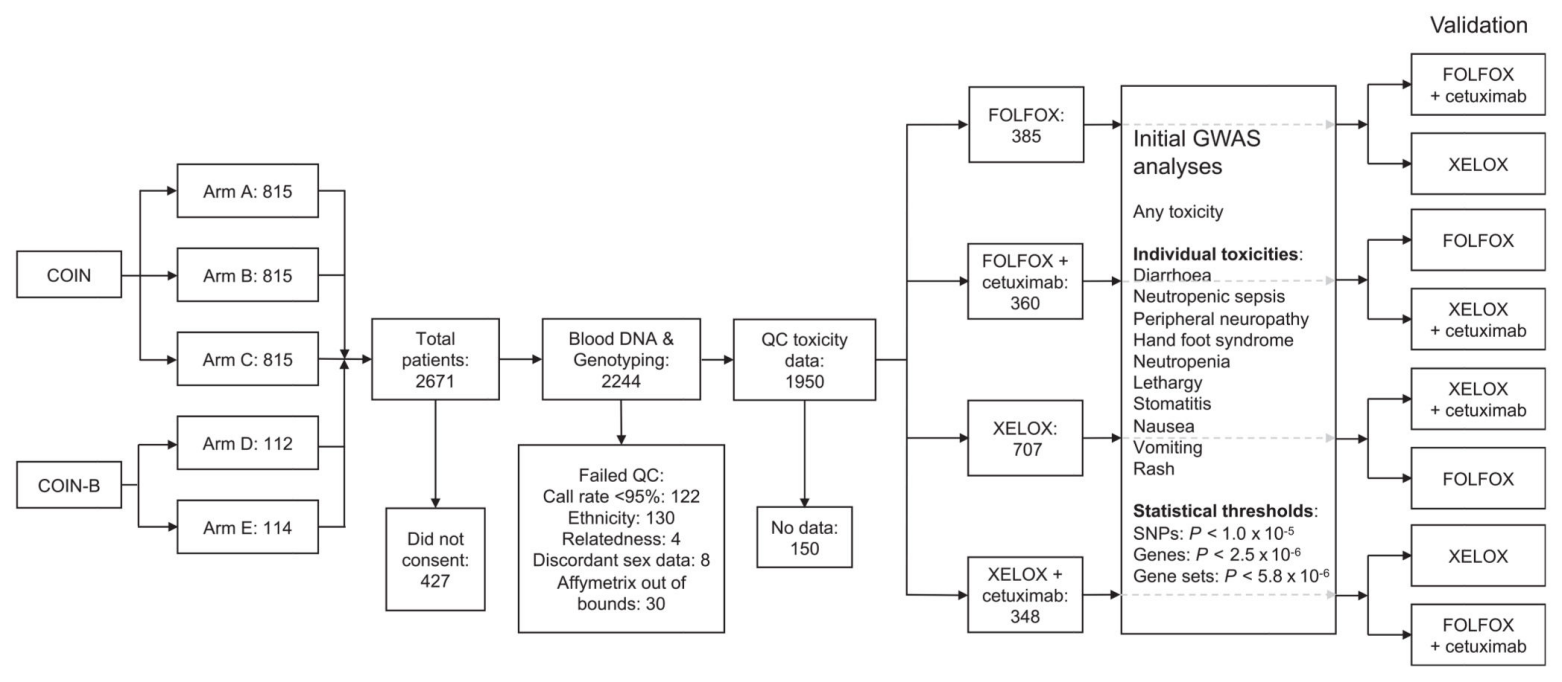
CONSORT diagram of the analysis strategy. COIN patients were randomised 1:1:1 to receive continuous oxaliplatin and fluoropyrimidine chemotherapy (Arm A, n = 815), continuous chemotherapy with cetuximab (Arm B, n = 815), or intermittent chemotherapy (Arm C, n = 815). COIN-B patients were randomised 1:1 to receive intermittent chemotherapy and cetuximab (Arm D, n = 112) or intermittent chemotherapy and continuous cetuximab (Arm E, n = 114). Of these, 2244 were genotyped on Axiom arrays, 1950 passed genotyping quality control (QC) and 1800 were segregated into groups according to chemotherapy regimen and cetuximab status (385 patients received FOLFOX, 360 FOLFOX + cetuximab, 707 XELOX and 348 XELOX + cetuximab). We conducted genome-wide association studies for any toxicity and 10 individual toxicities together with gene and gene set analyses. Single nucleotide polymorphisms (SNPs), genes and gene sets that reached genome-wide or suggestive significance were independently validated in the COIN and COIN-B group with the same chemotherapy regimen but alternative cetuximab status, and the COIN and COIN-B group with the alternative chemotherapy regimen but with the same cetuximab status

In total, 2671 patients with metastatic or locally advanced colorectal adenocarcinoma were recruited into the MRC clinical trials COIN (ISRCTN27286448)^16,17^ and COIN-B (ISRCTN3837568).^18^ None of the patients had previously received chemotherapy for advanced disease. COIN patients were randomised 1:1:1 to receive continuous oxaliplatin and fluoropyrimidine chemotherapy (Arm A, n = 815), continuous chemotherapy with cetuximab (Arm B, n = 815) or intermittent chemotherapy (Arm C, n = 815). COIN-B patients were randomised 1:1 to receive intermittent chemotherapy and cetuximab (Arm D, n = 112) or intermittent chemotherapy and continuous cetuximab (Arm E, n = 114) (Figure 1). For the first 12 weeks, treatments were identical in all patients apart from the choice of fluoropyrimidine (n = 1068, 40% received FOLFOX and n = 1603, 60% received XELOX) together with the randomisation of ± cetuximab (n = 1041, 39% received cetuximab) (Figure 1). Overall, patients had a mean age at randomisation of 62 years (range, 18-87) and 36% were female. Blood DNA samples were prepared from 2244 of the 2671 patients.

### 2.2 Clinical end points assessed and power considerations

Assessment of toxicity was performed at 12 weeks, since at this point patients from all trial arms received identical levels of chemotherapy (choice of XELOX or FOLFOX) with or without cetuximab. This time point was also prior to any interruption to treatment for the intermittent therapy arms.

The primary end point assessed was any toxicity graded by critical adverse events as per the Common Terminology Criteria for Adverse Events (CTCAE version 4.0) with the highest grade noted within the first 12 weeks of treatment (assessed at 6 and 12 weeks). Secondary end points were individual toxicities (diarrhoea, neutropenic sepsis, peripheral neuropathy, hand-foot syndrome, neutropenia, lethargy, stomatitis, nausea, vomiting and rash) graded by CTCAE score with the highest grade noted within the first 12 weeks of treatment (assessed at 6 and 12 weeks). Patients with toxicities graded 2 to 5 were compared against those graded 0 to 1.

Logistic regression models were used to determine if the chemotherapy regimen and cetuximab administration affected toxicity occurrence. Power to detect the toxicity effect sizes was calculated using the *genpwr* package in R,^19^ based upon 70% power, a standard GWAS significance of *P* = 5.0 × 10^−8^ and single nucleotide polymorphisms (SNPs) with minor allele frequencies (MAFs) of 0.20.

### 2.3 Genotyping

In all, 2244 patients were genotyped using Affymetrix Axiom Arrays according to the manufacturer’s recommendations (Affymetrix, Santa Clara, CA) at the King Faisal Specialist Hospital and Research Center, Saudi Arabia (under IRB approval 2110033).^20^ After quality control, SNP genotypes were available for 1950 patients.^20^ For 150 of the 1950 patients, no data on toxicity had been collected at 12 weeks and these were excluded leaving 1800 for analysis (Figure 1). Prediction of untyped SNPs was carried out using IMPUTEv2 (v2.3.0) based on data from the 1000 Genomes Project (Phase 1, December 2013) as reference. Additional imputation was performed for an 800 Mb region surrounding *MROH5* (to provide better SNP coverage) using the Phase 3 1000 Genome Project as reference. We restricted our analysis to directly typed SNPs and imputed SNPs with INFO scores of ≥0.8, a Hardy-Weinberg equilibrium of ≥1.0 × 10^−6^ and a MAF of ≥0.05.

### 2.4 Initial GWAS analyses

Patients from COIN and COIN-B were analysed for associated genetic biomarkers after segregating by chemotherapy regimen and cetuximab status; 385 patients had FOLFOX, 360 had FOLFOX + cetuximab, 707 had XELOX and 348 had XELOX + cetuximab (Figure 1). Genome-wide association analyses were run under a univariate additive model in Plink v1.9^21^ and results were plotted in R studio using qqman.^22^ A logistic regression method was chosen. SNPs that showed an association at *P* < 1.0 × 10^−5^ (suggestive of significance) were selected for independent validation. Results are reported in accordance with STREGA guidelines.

### 2.5 MAGMA gene and gene set analyses

MAGMA^23^ was used for gene and gene set analyses using data files from the NCBI 37.3 gene definitions and ∼8500 predefined gene sets. Gene analyses were run under a snpwise univariate model imposing a Bonferroni corrected significance threshold of *P* = 2.5 × 10^−6^ (Figure 1). Gene set analyses were run under both competitive and self-contained models with a corrected significance threshold of *P* = 5.8 × 10^−6^ (Figure 1).

### 2.6 Validation analyses

SNPs, genes and gene sets that reached genome-wide or suggestive significance in the GWAS analyses were independently validated in: (a) the COIN and COIN-B group with the same chemotherapy regimen but alternative cetuximab status and (b) the COIN and COIN-B group with the alternative chemotherapy regimen but with the same cetuximab status (Figure 1). For example, a SNP identified from the group receiving FOLFOX was validated in those receiving FOLFOX + cetuximab and in those receiving XELOX. A SNP identified from the group receiving XELOX was validated in those receiving XELOX + cetuximab and in those receiving FOLFOX. A SNP identified from the group receiving FOLFOX + cetuximab was validated in those in receiving FOLFOX and those receiving XELOX + cetuximab. A SNP identified from the group receiving XELOX + cetuximab was validated in those in receiving XELOX and those receiving FOLFOX + cetuximab (Figure 1). We considered a nominally significant threshold of *P* < .05 as evidence for validations. We had >85% power to detect our initially observed odds ratios for each validation subgroup.

Because rs13260246-reached genome-wide significance for vomiting in patients treated with XELOX, we also sought validation for this biomarker using data from 927 patients enrolled in the Quick and Simple and Reliable trial (QUASAR2). This was an open-label randomised Phase 3 clinical trial of capecitabine or capecitabine plus bevacizumab in patients with Stage II or III CRCs.^24^ Patients were genotyped using the Illumina genome-wide SNP panels (Human Hap 370, Human Hap 610 or Human Omni 2.5). Imputation was performed using IMPUTEv2 with 1000 genomes as reference. The INFO score for rs13260246 was 0.96. Vomiting was graded using the CTCAE scale and patients with grades 2 to 5 (22%) were compared to those with grades 0 to 1.

### 2.7 Bioinformatic analyses

The Genotype-Tissue Expression project database was used to identify expression quantitative trait loci (eQTLs) and splicing quantitative trait loci (sQTLs) for relevant SNPs (https://gtexportal.org/home). Significance for tissue association was set at *P* < 1.0 × 10^−3^ (ie, Bonferroni correction for 49 tissues [0.05/49]). Fine-mapping was used for SNPs at validated loci; conditional regression was first used to identify the number of causal variants and fine-mapping was then run using PAINTOR,^25^ which employs a Bayesian permutation method incorporating ENCODE and FANTOM5 functional annotations. Credible sets of causal SNPs were assembled for 95% coverage.

## 3 Results

There were significant differences in the incidences of toxicities associated with different chemotherapy regimens and cetuximab administration in COIN and COIN-B (Table 1; Supplementary Table 1). Notably, patients treated with FOLFOX had a significantly higher incidence of neutropenic sepsis, neutropenia and stomatitis, those with XELOX had a higher incidence of nausea and those with cetuximab had a higher incidence of skin rash, hand-foot syndrome and diarrhoea (Table 1). In view of this, patients were analysed for associations with genetic biomarkers after segregation by chemotherapy treatment and cetuximab status (Figure 1). There were no clinicopathological differences between these treatment groups (Supplementary Table 2).

In total, 4 million SNPs were analysed for a relationship with any toxicity and 10 individual toxicities in each of the four patient groups. Q-Q plots of observed vs expected *χ*^2^-test statistics showed no evidence for an inflation of test statistics for all 40 GWAS’s performed (λ range, 0.99-1.02) (Supplementary Figure 1). We had 70% power to detect a mean OR of 4.3 (range, 3-6) for any toxicity and 5.9 (2-39) for individual toxicities (Supplementary Table 3).

### 3.1 Relationship between SNP genotype and any toxicity

**Figure 2:**
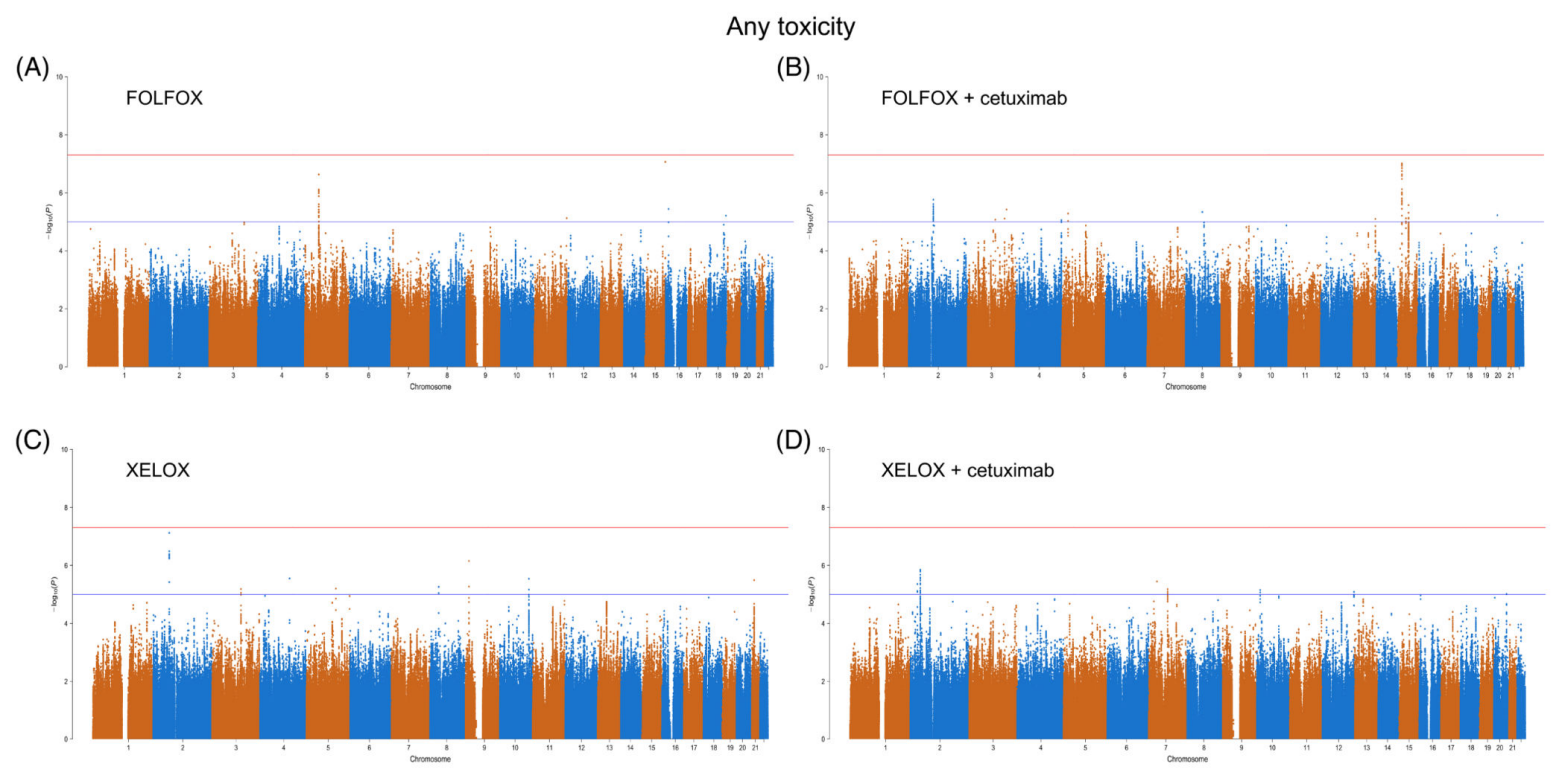
Manhattan plots of the relationship between single nucleotide polymorphism (SNP) genotype and any toxicity. Patients treated with (A) FOLFOX (n = 385), (B) FOLFOX + cetuximab (n = 360), (C) XELOX (n = 707) and (D) XELOX + cetuximab (n = 348). The red line indicates a genome-wide significance threshold of *P* = 5.0 × 10^−8^ and the blue line indicates a suggestive significance threshold of *P* = 1.0 × 10^−5^ [Color figure can be viewed at wileyonlinelibrary.com]

No SNPs were associated with any toxicity at genome-wide significant levels (*P* < 5.0 × 10^−8^). SNPs at 27 loci were associated at suggestive levels (*P* < 1.0 × 10^−5^) (5 with FOLFOX, 8 with FOLFOX + cetuximab, 7 with XELOX and 7 with XELOX + cetuximab) (Figure 2); however, no lead SNPs were independently validated in COIN and COIN-B patients treated with the same chemotherapy regimen but alternative cetuximab status, or alternative chemotherapy regimen but with the same cetuximab status, despite having >85% power (Supplementary Table 4).

### 3.2 Relationship between SNP genotype and individual toxicity

#### 3.2.1 Vomiting

**Figure 3:**
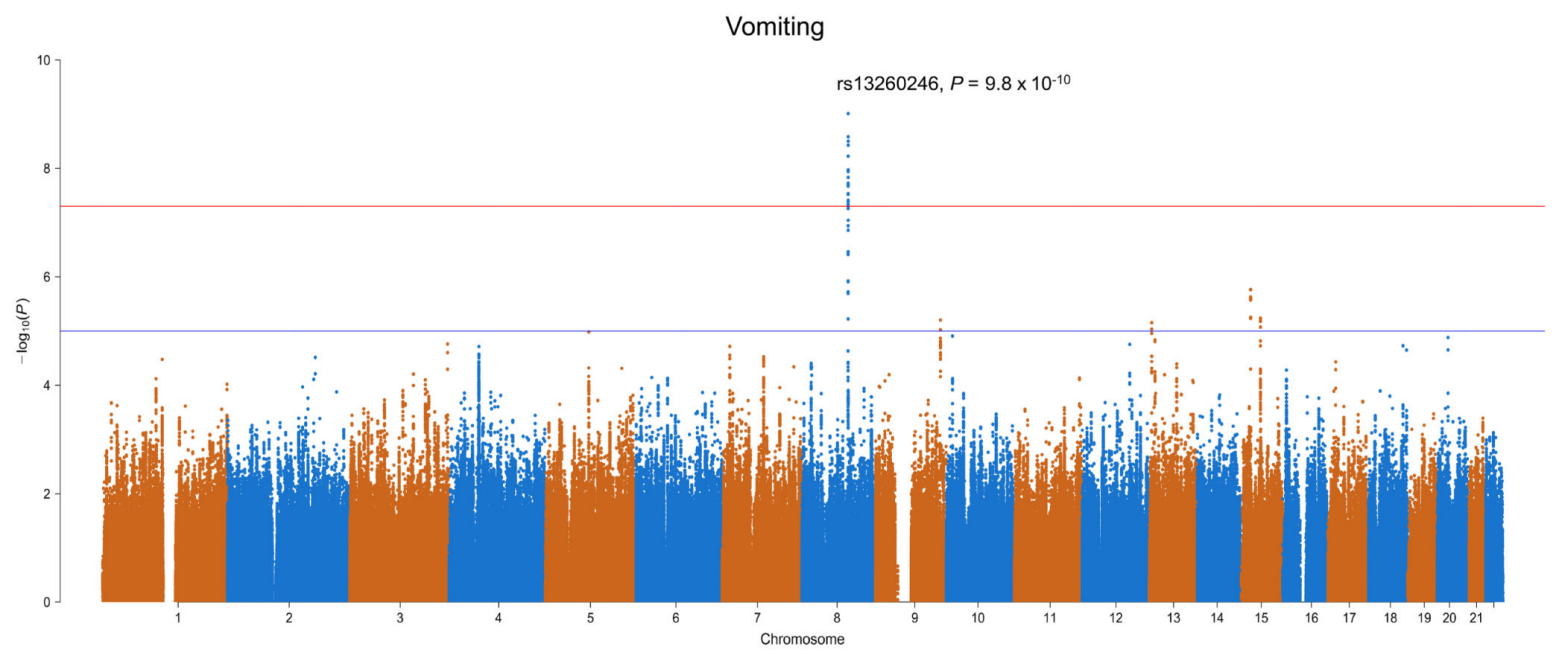
Manhattan plot of the association between single nucleotide polymorphism (SNP) genotype and vomiting in patients treated with XELOX. The red line corresponds to a *P* = 5.0 × 10^−8^ and the blue line *P* = 1.0 × 10^−5^ [Color figure can be viewed at wileyonlinelibrary.com]

rs13260246 at 8q21.3 was significantly associated with vomiting in patients treated with XELOX (odds ratio [OR] = 5.0, 95% confidence intervals [CIs] = 3.0-8.3, *P* = 9.8 × 10^−10^; Figure 3). However, the association was not validated in COIN and COIN-B patients treated with XELOX + cetuximab (*P* = .72), nor in those receiving FOLFOX (*P* = .35), with >90% power (Supplementary Table 5). We also failed to validate the association for rs13260246 with vomiting in the QUASAR2 trial of capecitabine alone vs capecitabine + bevacizumab for Stage II and III CRC, regardless of treatment arm studied (with >99% power) (Supplementary Table 5). rs13260246 was an eQTL for *SLC26A7* and five other genes (Supplementary Figure 2). SNPs at 15 loci had suggestive associations with vomiting but none were independently validated.

#### 3.2.2 Diarrhoea

SNPs at 21 loci had suggestive associations with diarrhoea (Supplementary Figure 3); however, only rs6030266 at 20q13.12 in patients treated with XELOX + cetuximab (OR = 0.4, 95% CI = 0.28-0.58, *P* = 5.7 × 10^−7^) was validated in patients receiving FOLFOX + cetuximab (OR = 0.7, 95% CI = 0.5-0.9, *P* = 3.6 × 10^−2^); pooled *P* = 3.2 × 10^−7^ (Table 2). rs6030266 maps to intron eight of the gene encoding protein tyrosine phosphatase receptor type T (*PTPRT*) (Supplementary Figure 4).

#### 3.2.3 Hand-foot syndrome

SNPs at 13 loci had suggestive associations with hand-foot syndrome (Supplementary Figure 3). Only rs1546161 at 1q21.2 in patients treated with FOLFOX (OR = 17.8, 95% CI = 5.1-62.0, *P* = 5.9 × 10^−6^) was validated in those receiving XELOX (OR = 1.7, 95% CI = 1.1-2.7, *P* = 2.5 × 10^−2^); pooled *P* = 2.5 × 10^−6^ (Table 2). rs1546161 maps to B-cell lymphoma 9 (*BCL9*) and was an eQTL for *GJA5* (Supplementary Figure 4).

#### 3.2.4 Neutropenia

SNPs at 13 loci had suggestive associations with neutropenia (Supplementary Figure 3). Only rs9601722 at 13q31.1 in patients treated with FOLFOX + cetuximab (OR = 3.4, 95% CI = 2.0-5.7, *P* = 5.2 × 10^−6^) was independently validated in those receiving FOLFOX (OR = 1.7, 95% CI = 1.1-2.9, *P* = 3.6 × 10^−2^); pooled *P* = 3.0 × 10^−6^ (Table 2). rs9601722 maps to a lncRNA (*LOC105370284*).

#### 3.2.5 Lethargy

SNPs at 12 loci had suggestive associations with lethargy (Supplementary Figure 3); however, only rs13413764 at 2q14.3 in patients treated with XELOX (OR = 1.8, 95% CI = 1.4-2.3, *P* = 4.5 × 10^−6^) was replicated in those receiving FOLFOX (OR = 1.5, 95% CI = 1.1-2.1, *P* = 9.2 × 10^−3^); pooled *P* = 7.5 × 10^−7^ (Table 2). rs13413764 maps to an intergenic region.

#### 3.2.6 Nausea

SNPs at 12 loci had suggestive associations with nausea (Supplementary Figure 3). However, only rs4600090 at 1p33 in patients treated with FOLFOX + cetuximab (OR = 4.0, 95% CI = 2.2-7.2, *P* = 5.9 × 10^−6^) was independently validated in those receiving FOLFOX (OR = 2.0, 95% CI = 1.1-4.0, *P* = 4.2 × 10^−2^); pooled *P* = 4.0 × 10^−6^ (Table 2). rs4600090 was an eQTL for *CMPK1, FOXE3* and *PDZK1IP1* (Supplementary Figure 4).

#### 3.2.7 Peripheral neuropathy, stomatitis, rash and neutropenic sepsis

SNPs at 15, 10, 8 and 4 loci had suggestive associations with peripheral neuropathy, stomatitis, skin rash and neutropenic sepsis, respectively, but no lead SNPs were independently validated.

### 3.3 MAGMA gene and pathway analyses

Gene and pathway analyses were performed considering approximately 17 000 genes and 8500 gene sets. Four genes were significantly associated with neutropenia (using a Bonferroni corrected threshold of *P* < 2.5 × 10^−6^). Of these, Maestro Heat-Like Repeat Family Member 5 (*MROH5*), found in patients treated with XELOX (*P* = 6.6 × 10^−7^), was independently validated in those receiving XELOX + cetuximab (*P* = 3.3 × 10^−2^); pooled *P* = 3.7 × 10^−7^ (Table 3; Supplementary Figure 5). Under a multivariate model accounting for sex and age, *MROH5* remained significant in a pooled analysis of patients treated with XELOX and XELOX + cetuximab; pooled *P* = 1.0 × 10^−6^.

*MROH5* lies at 8q24.3, one of the 13 loci of suggestive association with neutropenia. The association of *MROH5* with neutropenia appeared to be due to independent sets of SNPs in patients treated with XELOX (lead SNP rs76380775 OR = 4.8, 95% CI = 2.4-9.5, *P* = 1.4 × 10^−6^) as compared to those receiving XELOX + cetuximab (lead SNP rs12056882 OR = 4.4, 95% CI = 1.4-14, *P* = 1.0 × 10^−2^; Supplementary Figure 6). Neither rs76380775 nor rs12056882 was associated with neutropenic sepsis or white blood cell count. rs12056882 was a sQTL for *PTP4A3* (which lies 1.37 kb downstream of *MROH5*).

One gene was significantly associated with stomatitis, 3 genes (all mapping to 8q21.3) were associated with vomiting (Table 3) and 4, 8 and 3 gene sets were associated with any toxicity, lethargy and vomiting, respectively; however, all failed independent validation (Supplementary Tables 6 and 7).

### 3.4 Lack of confounding effect for rare *DPYD* variants

We have previously shown that two rare variants in *DPYD* (Asp949Val and IVS14+1G>A) were associated with a range of toxicities in COIN and COIN-B.^15^ Of the 1800 patients in our current GWASs, 22 carried Asp949Val and 17 carried IVS14 +1G>A. Excluding these patients made no significant differences to the strengths of associations reported *herein* (Supplementary Table 8).

### 3.5 Alternative model of toxicity

We considered an alternative model of toxicity comparing patients with grades 3 to 5 (ie, severe toxicity) to patients with grades 0 to 2 (no, mild or moderate toxicity) for all biomarkers identified *herein* (Supplementary Table 9). Five of the seven biomarkers remained nominally significant.

### 3.6 Evaluation of previously purported associations

A previous GWAS for toxicity to 5-FU or FOLFOX in patients with CRC identified two SNPs associated with mucositis, two with diarrhoea and three with haematological toxicities, albeit only at nominal significance.^26^ We failed to validate any of these SNPs in COIN and COIN-B (Supplementary Table 10), despite having adequate power.

## 4 Discussion

*MROH5* was identified from MAGMA gene analyses as associated with neutropenia at genome-wide significant levels in patients treated with XELOX and was independently validated in those receiving XELOX + cetuximab. Interestingly, this association appeared to be due to independent sets of SNPs in these two patient groups and rs12056882 was a sQTL for *PTP4A3* which lies adjacent to *MROH5. MROH5* has been suggested to be both a pseudogene and a functional gene (with an unknown role) dependent upon the status of a SNP that introduces a premature termination codon. *PTP4A3* represents a strong causal candidate for neutropenia as treatment of mice with a *PTP4A3* derived peptide reduced endotoxemia-induced septic shock.^27^ *PTP4A3* expression has also been associated with poor prognosis in CRC possibly due to a role in metastasis and tumour invasion,^28,29^ and has been implicated in resistance to chemotherapy.^30,31^ Importantly, the strength of the relationship between SNPs in *MROH5* and neutropenia suggests that they may have clinical utility as predictive biomarkers.

We also found a clear signal for rs13260246 associated with vomiting in patients treated with XELOX. However, this association was not validated in patients treated with XELOX + cetuximab, nor in those receiving FOLFOX, nor in patients treated with capecitabine ± bevacizumab from the QUASAR2 trial. Given that we had sufficient power to replicate the initial observation, these data suggest that rs13260246 is a false-positive although it remains possible that the association with vomiting is specific to those treated with XELOX alone. rs13260246 maps to, and is an eQTL for, *SLC26A7*, which functions as a Cl^−^/HCO_3_^−^ exchanger and chloride channel,^32^ and is expressed in several tissues including the thyroid. Chemotherapy can cause thyroid dysfunction and response to treatment may be affected by pre-existing thyroid conditions.^33–35^ *SLC26A7* is also expressed in parietal cells and genetic deletion results in decreased gastric acid secretion.^36,37^ Both thyroid and gastric dysfunction can cause vomiting.^38,39^ Therefore, *SLC26A7* represents a strong biological candidate for vomiting, but lacks genetic validation.

In total, we found SNPs at 139 loci with evidence for suggestive associations for any toxicity or individual toxicities and lead SNPs at five of these were validated at nominally significant levels. However, if we applied a more stringent correction for 139 validation tests, none of the five would have passed the adjusted significance threshold. Further validation of these biomarkers in independent cohorts is therefore necessary before they could be applied in clinical practice. rs6030266 was associated with diarrhoea and identified in patients treated with cetuximab. It maps to intron eight of *PTPRT*, a tumour suppressor gene that functions as part of the JAK/STAT pathway.^40^ rs1546161 was associated with hand-foot syndrome and maps to *BCL9*, overexpression of which has been linked to disrupted *wnt* signalling.^41^ rs1546161 is also an eQTL for *GJA5*, a gap junction protein with significant expression in subcutaneous adipose tissue. rs4600090 associated with nausea lies within and is an eQTL for *CMPK1*, an enzyme associated with activation of 5-FU phosphorylation and linked to 5-FU sensitivity.^42^ rs4600090 is also an eQTL for *PDZK1IP1* which functions as a cargo protein expressed in the adrenal glands. Interestingly, noradrenaline and cortisol, hormones released by adrenal glands, have both been associated with chemotherapy-induced nausea.^43^ rs9601722 associated with neutropenia and rs13413764 with lethargy did not lie within protein coding gene regions.

Our study had limited power to detect common variants associated with toxicity with low odds ratios (<2) and our attempts to validate any findings were limited by groups with similar, but non-identical, therapies. Nonetheless, after conducting 40 GWASs on large patient cohorts with well-characterised clinical data, we conclude there is a lack of common variants with modest or large effect sizes associated with toxicities induced by oxaliplatin and fluoropyrimidine chemotherapy with or without cetuximab. In support of this, we failed to replicate loci previously suggested to be associated with toxicity to FOLFOX identified from another GWAS.^26^ Further analyses of *MROH5* and/or *PTP4A3* with neutropenia are warranted.

## Figures and Tables

**Table 1:** Patients with grades 2 to 5 CTCAE toxicities at 12 weeks

	FOLFOX treated			XELOX treated	
	n = 385 (%)	+ cetuximab n = 360 (%)		n = 707 (%)	+ cetuximab n = 348 (%)
Any toxicity	237 (61)	275 (76)		430 (61)	226 (65)
Individual toxicities					
Diarrhoea	78 (20)	109 (30)		165 (23)	123 (35)
Neutropenic sepsis	24 (8)	39 (16)		5 (0.7)	1 (0.3)
Peripheral neuropathy	43 (11)	30 (8)		110 (16)	44 (13)
Hand-foot syndrome	9 (2)	56 (16)		53 (8)	56 (16)
Neutropenia	100 (26)	119 (33)		36 (5)	6 (2)
Lethargy	130 (34)	126 (35)		258 (36)	103 (30)
Stomatitis	48 (12)	102 (28)		32 (5)	29 (8)
Nausea	41 (11)	47 (13)		142 (20)	68 (20)
Vomiting	25 (6)	34 (9)		87 (12)	35 (10)
Rash	5 (1)	196 (54)		11 (2)	166 (48)

*Note:* Percentage of patients in parentheses. We had 70% power to detect a mean OR of 4.3 (range, 3-6) for any toxicity and 5.9 (2-39) for individual toxicities (Supplementary Table 3). For neutropenic sepsis in patients treated with XELOX and XELOX + cetuximab, neutropenia in patients treated with XELOX + cetuximab and rash in patients treated with FOLFOX, we had insufficient power to perform the genome-wide association studies (GWASs); therefore, in total, we conducted 40 GWASs.

**Table 2:** Validated single nucleotide polymorphisms (SNPs) associated with individual toxicities

Toxicity	Treatment group	Lead SNP	Cytoband	Initial GWAS				Validation chemo		Validation cetuximab status		Combined
				OR	95% CI	*P*-value		*P*-value		*P*-value		*P*-value
Diarrhoea	XELOX + cetuximab	rs6030266	20q13.12	0.4	0.3-0.6	5.7 × 10^−7^		.33		3.6 × 10^−2^		3.2 × 10^−7^
Hand-foot syndrome	FOLFOX	rs1546161	1q21.2	17.8	5.1-62	5.9 × 10^−6^		.13		2.5 × 10^−2^		2.5 × 10^−6^
Neutropenia	FOLFOX + cetuximab	rs9601722	13q31.1	3.4	2.0-5.7	5.2 × 10^−6^		3.6 × 10^−2^		NA		3.0 × 10^−6^
Lethargy	XELOX	rs13413764	2q14.3	1.8	1.4-2.3	4.5 × 10^−6^		NA		9.2 × 10^−^ ^3^		7.5 × 10^−7^
Nausea	FOLFOX + cetuximab	rs4600090	1p33	4.0	2.2-7.2	5.9 × 10^−6^		4.2 × 10^−2^		.55		4.0 × 10^−6^

Abbreviations: CI, confidence intervals; Combined, pooled *P*-value of initial GWAS cohort and validated cohort (excludes cohort which was not validated); NA, OR in the opposite direction to the initial GWAS; OR, odds ratio; Validation cetuximab status, validation in the COIN and COIN-B group with the alternative chemotherapy regimen but with the same cetuximab status; Validation chemo, validation in the COIN and COIN-B group with the same chemotherapy regimen but alternative cetuximab status.

**Table 3:** MAGMA gene analyses for individual toxicities

Toxicity	Treatment group	Gene	*P*-value	Validation chemo *P*-value	Validation cetuximab status *P*-value	Pooled *P*-value
Neutropenia	FOLFOX	*RPL17-C18orf32*	8.9 × 10^−7^	.57	.53	—
		*C18orf32*	1.3 × 10^−6^	.56	.51	—
		*RPL17*	1.5 × 10^−6^	.56	.52	—
	XELOX	***MROH5***	**6.6 × 10** ^−**7**^	**3.3 × 10** ^−**2**^	**.09**	**3.7 × 10** ^−**7**^
Stomatitis	FOLFOX	*SCAF4*	1.3 × 10^−6^	.07	.61	—
Vomiting	XELOX	*LRRC69*	1.2 × 10^−7^	.77	.73	—
		*SLC26A7*	4.3 × 10^−7^	.81	.60	—
		*PIP4P2*	9.7 × 10^−7^	.94	.34	—

*Note:* Significance was set at a Bonferroni-corrected significance threshold of *P* < 2.5 × 10^−6^. Only *MROH5* was significantly associated with neutropenia in patients treated with XELOX and was independently validated in patients receiving XELOX + cetuximab (*P* = 3.3 × 10^−2^), with a pooled *P* = 3.7 × 10^−7^ (in bold) (and *P* = 5.8 × 10^−7^ when also including the FOLFOX cohort). Abbreviations: Validation cetuximab status, Validation in the COIN and COIN-B group with the alternative chemotherapy regimen but with the same cetuximab status; Validation chemo, validation in the COIN and COIN-B group with the same chemotherapy regimen but alternative cetuximab status.

## Data Availability

The GWAS summary statistics are available through the NHGRI-EBI GWAS Catalog under study accession numbers GCST90017191 - GCST90017231: http://ftp.ebi.ac.uk/pub/databases/gwas/summary_statistics/GCST90017001-GCST90018000. Further details and other data that support the findings of this study are available from the corresponding author upon request.

## What’s new?

Among cancer patients, toxic side effects of chemotherapeutic agents can vary considerably. This inter-individual variability may be influenced by genetic factors. Here, genome-wide association studies were analysed for toxicities to oxaliplatin and fluoropyrimidine chemotherapy (FOLFOX and XELOX) in patients with advanced colorectal cancer. In XELOX-treated patients, *MROH5* variants were strongly associated with neutropenia. The variant rs13260246, mapped to *SLC26A7*, was associated with vomiting. Suggestive associations for toxicities were identified for single nucleotide polymorphisms (SNPs) at 139 loci; 5 of which validated. The validated variants could serve as predictive biomarkers for specific chemotherapy-related toxicities.

## Abbreviations

CRC: colorectal cancer
CTCAE: Common Terminology Criteria for Adverse Events
eQTL: expression quantitative trait loci
GWAS: genome-wide association study
QUASAR2: Quick and Simple and Reliable Trial
SNP: single nucleotide polymorphism
sQTL: splicing quantitative trait loci.
